# High-Resolution Analysis of Growth and Transpiration of Quinoa Under Saline Conditions

**DOI:** 10.3389/fpls.2021.634311

**Published:** 2021-08-05

**Authors:** Viviana Jaramillo Roman, Rick van de Zedde, Joseph Peller, Richard G. F. Visser, C. Gerard van der Linden, Eibertus N. van Loo

**Affiliations:** ^1^Plant Breeding, Wageningen University and Research, Wageningen, Netherlands; ^2^Graduate School Experimental Plant Sciences, Wageningen University, Wageningen, Netherlands; ^3^Wageningen Plant Research, Wageningen, Netherlands

**Keywords:** quinoa, salt stress, Plantarray, transpiration, stomatal conductance, water use efficiency, radiation use efficiency, phenotyping

## Abstract

The Plantarray 3.0 phenotyping platform^®^ was used to monitor the growth and water use of the quinoa varieties Pasto and selRiobamba under salinity (0–300 mM NaCl). Salinity reduced the cumulative transpiration of both varieties by 60% at 200 mM NaCl and by 75 and 82% at 300 mM NaCl for selRiobamba and Pasto, respectively. Stomatal conductance was reduced by salinity, but at 200 mM NaCl Pasto showed a lower reduction (15%) than selRiobamba (35%), along with decreased specific leaf area. Diurnal changes in water use parameters indicate that under salt stress, daily transpiration in quinoa is less responsive to changes in light irradiance, and stomatal conductance is modulated to maximize CO_2_ uptake and minimize water loss following the changes in VPD (vapor pressure deficit). These changes might contribute to the enhanced water use efficiency of both varieties under salt stress. The mechanistic crop model LINTUL was used to integrate physiological responses into the radiation use efficiency of the plants (RUE), which was more reduced in Pasto than selRiobamba under salinity. By the end of the experiment (eleven weeks after sowing, six weeks after stress), the growth of Pasto was significantly lower than selRiobamba, fresh biomass was 50 and 35% reduced at 200 mM and 70 and 50% reduced at 300 mM NaCl for Pasto and selRiobamba, respectively. We argue that contrasting water management strategies can at least partly explain the differences in salt tolerance between Pasto and selRiobamba. Pasto adopted a “conservative-growth” strategy, saving water at the expense of growth, while selRiobamba used an “acquisitive-growth” strategy, maximizing growth in spite of the stress. The implementation of high-resolution phenotyping could help to dissect these complex growth traits that might be novel breeding targets for abiotic stress tolerance.

## Introduction

Plant breeding for abiotic stress tolerance has proven to be complex (Gilliham et al., 2017). A major challenge is that stress tolerance is a systemic process that involves a number of synchronized, interconnected physiological processes and genes operating together. A second important complication is that these physiological processes are largely and continuously influenced by the environment. Thus, a proper screening of tolerance traits would ideally involve continuous monitoring of the plant responses to changes in the environment, which means that an accurate physiological phenotyping of well-defined traits is essential for the successful breeding for salt tolerance. Plant phenotyping has rapidly evolved in the past decades and has benefited enormously from developments in other disciplines such as remote sensing, robotics, computer vision and machine learning (Furbank and Tester, 2011). Most state-of-the-art phenotyping facilities, particularly for stress-related traits, collect information using robotics and automated image acquisition and analysis: image-based phenotyping (Fahlgren et al., 2015). A complementary platform implements physiology-based gravimetric systems that enable the direct measurement of plant dynamic responses, also called functional phenotyping (Negin and Moshelion, 2017). The data provided by gravimetric platforms, together with controlled measurements of environmental parameters, such as radiation, humidity, atmospheric vapour-pressure deficit (VPD) and temperature provide new insights into the complex genotype x environment interactions under specific treatments or abiotic stresses (Negin and Moshelion, 2017).

Soil salinization is a major limiting factor for agriculture, causing significant pressure on the availability of arable land. Saline soils constitute more than 20% of the global irrigated land and affect agricultural production in more than 75 countries. Soil salinity causes severe yield and economic losses, especially to smallholder farmers worldwide, and is expected to expand as a result of climate change (Qadir et al., 2014). Plant growth is directly and indirectly affected by soil salinity. Growth takes place by the conversion of photosynthates into structural molecules. Energy from photosynthesis is also needed to maintain several physiological functions that rely on assimilation of carbon dioxide and glucose metabolism, known as maintenance respiration (De Vries, 1975). When salt accumulates in the soil, the osmotic potential decreases, and the osmotic gradient between root medium and the roots leads to reduced water uptake with a subsequent reduction in cell expansion (Munns, 2002). By adjusting internal osmotic potential by for instance the accumulation of inorganic and/or organic compounds, plants can restore water uptake, at least up to a certain degree. The salinity-induced water uptake limitation directly affects growth through decreased CO_2_ availability resulting from stomata closure and down-regulation of photosynthetic metabolism (Chaves et al., 2008). The indirect effects of salt stress on growth include possible damage to the photosynthetic machinery caused by the secondary oxidative stress prompted by salinity as well as an increased maintenance respiration caused by several costly salt stress response mechanisms (i.e., osmotic adjustment, ion transport) (Karlberg et al., 2006). Due to the increased maintenance respiration less assimilates will be available for plant growth with the same amount of transpired water, which would lead to a decreased water use efficiency (Munns et al., 2020).

The assessment of plant growth and the relation with transpiration and transpiration efficiency would provide a mechanistic account of the salinity effects at the whole plant level. However, transpiration has always been a trait that is laborious and expensive to measure and this has limited its incorporation in salt tolerance studies. Therefore, we only have fragmented understanding of the consequences of salinity-induced changes in transpiration on growth and yield reduction (Harris et al., 2010). In the present study we use a functional phenotyping platform based on mini-lysimeters, the Plantarray 3.0 platform (Plant-Ditech, Rehobot, Israel). This platform allows simultaneous and high temporal resolution measurements of water uptake, transpiration and plant growth to expand our understanding of salt stress responses of plants, using the facultative halophyte *Chenopodium quinoa* as a model species. *Chenopodium quinoa* is an herbaceous, annual crop that originated in the Andes and is well-adapted to harsh environments, such as nutrient-poor, drought-affected, and saline soils. The high salt tolerance of quinoa has been widely recognized (Hinojosa et al., 2018; Jaramillo Roman et al., 2020). The overall goals of this study are: (i) to explore the interacting effects of salt stress on water uptake, transpiration and growth of quinoa, (ii) to identify salt tolerance strategies of two quinoa genotypes known to differ in their response to salinity and (iii) to examine the potential of high-resolution functional phenotyping for identifying physiological markers for salt tolerance screening in breeding programs.

## Materials and Methods

### Plant Materials and Treatments

The European non-bitter quinoa varieties Pasto and selRiobamba, a line selected from Riobamba (Riobamba has still some residual heterozygosity) were used in this experiment. These varieties were bred at Plant Breeding, Wageningen University & Research (The Netherlands) and AbbottAgra (France) and in previous experiments they have shown contrasting responses to salt stress (Jaramillo Roman et al., 2020). The experiment was conducted between March and May 2019 at the Unifarm greenhouse facilities of Wageningen University & Research, The Netherlands. Plants were sown in trays filled with potting soil and transplanted to 4 L pots 16 days after sowing (DAS). The pots were filled with standard filtered sand (grain size 0.6–1.0 mm) and each pot contained 4 plants. To prevent evaporation, small PVC balls were put on the surface of the pots, surrounding the plants. The greenhouse air humidity was set to a minimum of 80% and the photoperiod to 16 h light. When the incoming shortwave radiation was below 200 Wm^–2^, additional lighting was supplied (100 Wm^–2^). The temperature in the greenhouse was set up to a minimum of 15°C during the night. During the day, ventilation was controlled so the temperature did not exceed 35°C. The plants were irrigated with half-concentrated Hoagland’s nutrient solution. Salt stress treatment started 33 DAS with irrigation with 0.5 × Hoagland’s solution plus 200 mM NaCl. However, due to the sudden increase to 200 mM NaCl salinity, wilting was observed in the leaves of treated plants a few hours after irrigation. Therefore, the excess of salt was washed out and the salt treatment was built up in incremental steps of 100 mM NaCl per day until the desired salt concentration was reached. The final salt treatments of 200 mM and 300 mM NaCl were reached on day 36 after sowing, and the soil salt concentration in the drainage was monitored continuously with a conductivity meter (Profile Cond 315i, Xylem Analytics, Germany) for the duration of the experiment. Four pots per variety were used in each treatment, each pot was considered one experimental unit. Half of the plants (2 plants per pot) were harvested 47 days after sowing and the remaining plants were harvested 77 days after sowing. During the first destructive harvest, the above-ground biomass was collected and separated into stems, leaves and inflorescences. Leaves were split in young (upper one-third of the plant) and old leaves (lower two-thirds of the plant). Fresh weights of leaves, stems and inflorescences were recorded, and leaf area was measured using a leaf area meter (Li-3000 Area Meter, Li-Cor, Lincoln, NE, United States). During the second destructive harvest, roots were collected as well and weighed. Dry weights were determined after drying the samples in a forced-air oven at 60°C until they reached stable weight. The salt tolerance index (STI) was calculated as the ratio of above-ground dry biomass of salt-treated plants and the dry biomass of control (0 mM NaCl) plants. A timetable of all the parameters assessed in this study is presented in Supplementary Table 1.

### Plantarray Design and Data Collection

**FIGURE 1:**
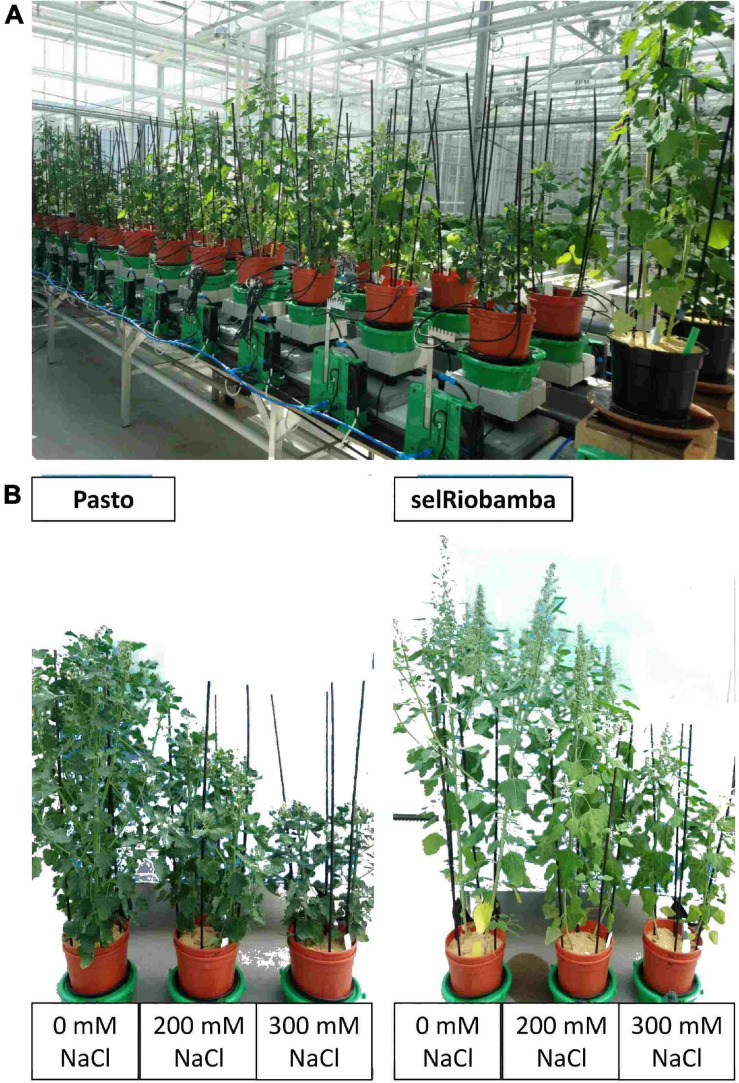
**(A)** Plant Array 3.0 platform used in this study. Each pot is positioned in a sensitive load cell connected to a control unit. **(B)** Pasto and selRiobamba at 77 DAS (6 weeks after the start of the salt treatment).

The functional phenotyping platform Plantarray 3.0 platform (Plant-Ditech, Rehobot, Israel) was used to monitor plant growth through controlled tracking and measuring of irrigation and biomass increase throughout the growing period. The system uses highly sensitive load cells that are used as weighing lysimeters. Additional sensors were incorporated to the system in order to monitor other environmental factors. These were: HC2-S3-L meteo probe for relative humidity and temperature in the greenhouse (Rotronic, Crawley, United Kingdom), LI-COR 190 Quantum Sensor for photosynthetically active radiation measurements (Lincoln, NE, United States), and a soil moisture, electro-conductivity and temperature sensor (5T, Decagon devices, Pullman, WA, United States) incorporated in every pot. We monitored environmental factors to calculate VPD throughout the experiment and to understand the influence of these environmental factors in the transpiration and other physiological parameter measured on the plants. Each load unit (containing one pot) was connected to an individual control unit (CR1000 data logger) (Campbell Scientific, Logan, UT, United States) (Figure 1A). The system recorded the weight of the pots plus the environmental information registered by the sensors every 3 min. The data collection could be viewed in real-time through the online web-based software SPAC-analytics (Plant Ditech, Rehobot, Israel). The physiological traits could not directly be extracted from the protocols implemented by the SPAC analytics software, because at the beginning of the experiments the seedlings were very small and despite the use of the PCV balls the effect of evaporation was considerable, and therefore the weight of the pots could not be equilibrated. Several pots containing only substrate were placed next to the system and weighed manually on a daily basis to estimate evaporation from the pots.

Additional pots were grown next to the system for the two varieties (8 pots with 4 plants each, each pot was considered one experimental unit) and harvested throughout the experiment for growth rate calculations. Several destructive harvests were performed on this material: (1) when seedlings were transplanted from trays to pots (16 days after sowing (DAS)), (2) when pots were incorporated to the system (26 DAS), (3) when the salt treatment started (36 DAS). The harvested material was used to measure leaf weight ratio (LWR) (g g^–1^), and specific leaf area (SLA) (m^2^ kg^–1^). SLA was calculated as the amount of leaf area per unit of leaf dry weight, LWR as the leaf fraction of the total dry plant biomass. RGR (d^–1^) was calculated as the natural logarithm of the relative increase in plant biomass over the mentioned period of time: RGR = ln(W_2_/W_1_)/(t_2_-t_1_) (Lambers and Poorter, 1992). Net assimilation rate (NAR, g m^–2^ day^–1^) was derived using the linear relation *RGR* = *LWR*×*SLA*×*NAR*. The RGR components calculated per period for the plants in the extra pots were used to estimate the RGRs of the plants in the system. To do so, it was assumed that the plants only grew during the light hours, and that RGR was strongly correlated with PAR during the day. This allowed us to derive RGRs for the plants in the system from the RGRs measured on the extra pots. Using the initial weight of the seedlings at the start of the experiment and assuming exponential growth of the plants, the derived RGRs were used to calculate fresh weights (FW) of the plants in the system with a 3-minute resolution. The measured fresh weights of the first three destructive harvest of the extra pots (16, 26 and 36 DAS) and the measured fresh weights on the plants on the system at 47 and 77 DAS were used as reference values to validate the calculation of FW values throughout the experiment.

The interpolated RGRs and FWs were used to estimate the other components of the RGR analysis (LWR and SLA) at each individual timepoint, and to obtain a reference value for the leaf area. Transpiration rate per time point equates rate of water loss from the pots, corrected for evaporation. This was calculated using the weight values of pots + plants provided by the system and subtracting the interpolated FW and the weight of static components added to the lead cells. Correction for evaporation was done based on the evaporation rates of the extra pots without plants. A running average of 180 min was used in the calculations to account for possible missing values in the weights provided by the lysimeters. Stomatal conductance (*gs*) was calculated as transpiration rate/leaf area/VPD. A running average of 120 minutes of data that was recorded every 3 min was used to correct for possible errors or outlier values in the system or VPD measurements. To validate the calculated *gs*, a portable leaf porometer (Decagon Devices Inc., WA, Australia) was used to measure*gs* on the abaxial side of the second fully developed non-shadowed leaf between 12:00 and 13:00 h at 58 DAS. Finally, whole plant water use efficiency (WUE) was calculated as g FW/g water transpired for an interval of 3 min in a 2 h running average using the interpolated fresh weights and transpiration rates.

### Integrating Phenotyping Data to a Crop Production Model

The mechanistic crop growth model LINTUL (Light interception and utilization) was used as a framework to integrate several physiological components to plant growth (Spitters and Schapendonk, 1990). LINTUL is based on the linear relationship between produced biomass and the amount of radiation intercepted by the crop. The crop growth rate is calculated as: d*W*_*t*_/*dt* = *f*_*t*_ = *PAR*_*t*_×*RUE*, whered*W*_*t*_/*dt* is the instantaneous growth rate at day t (g DM m^−2^ d^−1^), *PAR*_*t*_ the incoming amount of photosynthetically active radiation (MJ m^−2^ d^−1^, ‘light’ wave bands 400–700 nm), *f_t_* the fraction of PAR intercepted by the foliage, and RUE the average light utilization efficiency or radiation use efficiency (g DM MJ^−1^ PAR). The fraction of light intercepted during exponential growth can be calculated as 1^–exp(–*k LAI)*^ on the basis of simulated LAI, where LAI is the leaf area index (m^2^ leaf surface (W_t_ x LWR x SLA) m^−2^ ground surface) and k is the extinction coefficient (Spitters and Schapendonk, 1990). Based on several studies that applied the LINTUL crop growth model (Sinclair and Muchow, 1999), the radiation extinction coefficient (k) was assumed to be 0.8 for this experiment, and the area of the pot that intercepted light (based on a pot size of 40 cm × 60 cm) to be 1 m^2^. Following an Expo linear model, RUE can be related to the RGR through the following relations: 1/*W*_*t*_×*dW*_*t*_/*dt* = *RGR* = *LWR*×*SLA*×*NAR*, thus *NAR* = *PAR*_*t*_×*RUE* (Van Loo, 1992).

### Rapid Light Curve

Chlorophyll fluorescence measurements were performed at 76 DAS using the stand device Robin PSI PlantScreen TM system (Photon System Instruments, Brno, Czechia) for kinetic chlorophyll fluorescence analysis. The device is equipped with a chlorophyll fluorescence imaging unit FluorCam FC-800 mF Pulse Amplitude modulated (PAM). Three detached young leaves per plant were introduced in the device to perform the analysis. Rapid light curves were measured following 20 s acclimation at six different actinic light intensities (10-20-40-60-80-100% of a maximum actinic light of 1,692 μmol m^−2^ s^−1^) for a duration of 10 s. The calculated parameter was the PSII effective quantum yield (*φ_*PSII*_*) defined as (*F*’m–*F*’)/*F*’m where F’ is the fluorescence emission from a light-adapted leaf and F’m is the maximal efficiency from a light-adapted leaf. Relative electron transport rate (rETR) is an approximation of the rate of electrons pumped through the photosynthetic chain, and was estimated as: rETR = *φ_*PSII*_* x PAR × 0.85 × 0.5 where 0.85 is the value for absorption coefficient of the leaves and 0.5 the fraction of excitation energy distributed to PSII (Tschiersch et al., 2017).

### Thermal Imaging of Quinoa Leaves

A thermal camera (FLIR A655sc, FLIR Systems, INC., Wilsonville, United States) was mounted above the plants. This camera has a 640 × 480 pixels resolution, and a temperature range of −40°C to 150°C, with a spectral range of 7.5–14 μm. The camera was allowed to automatically perform a NUC calibration throughout the period of imaging (between day 58 and 66 after sowing). One picture frame was recorder every ten minutes and the leaf temperatures were measured in a circular region in the center of a young leaf of each plant as depicted in Figure 5A.

**FIGURE 2:**
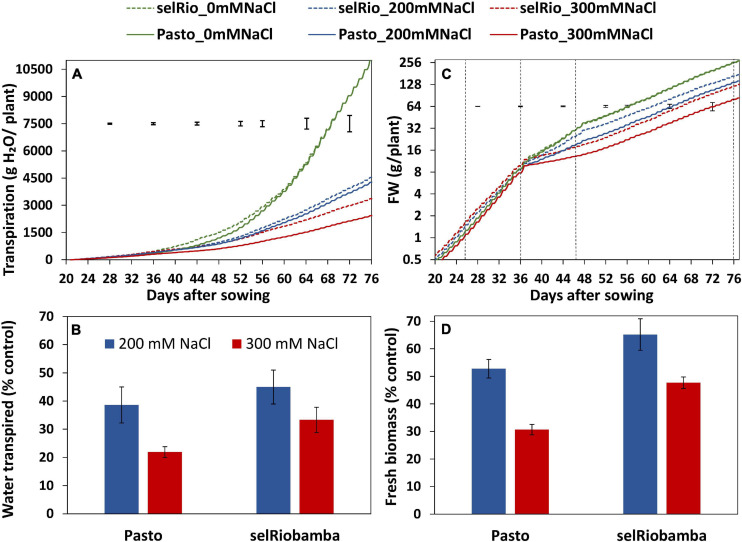
Dynamics of transpiration and growth throughout the season. **(A)** Cumulative plant transpiration in two varieties (Pasto and selRiobamba) and three salt concentrations (control (0 mM NaCl), 200 mM NaCl and 300 mM NaCl). **(B)** Total water transpired by the plants at 77 DAS under salt stress as a percentage of the control. **(C)** Fresh biomass per plant. Fresh weights were interpolated based on RGRs estimated from destructive harvest from extra pots (days 11, 21, and 36 after sowing) or plants growing in the system (47 and 77 DAS). The dotted black lines in the graph indicate the dates of the harvest in which the interpolated weights were validated with the biomass data from the harvests (16, 26, 36, 47, and 77 DAS). **(D)** Fresh biomass of the plants at 77 DAS under salt stress as a percentage of the controls. Means of 4 plants. In panels **(A,C)** the error bars indicate the SEM (standard error of mean) from the ANOVA at a specific timepoint. In panels **(B,D)** the error bars indicate the SE of the individual means.

**FIGURE 3:**
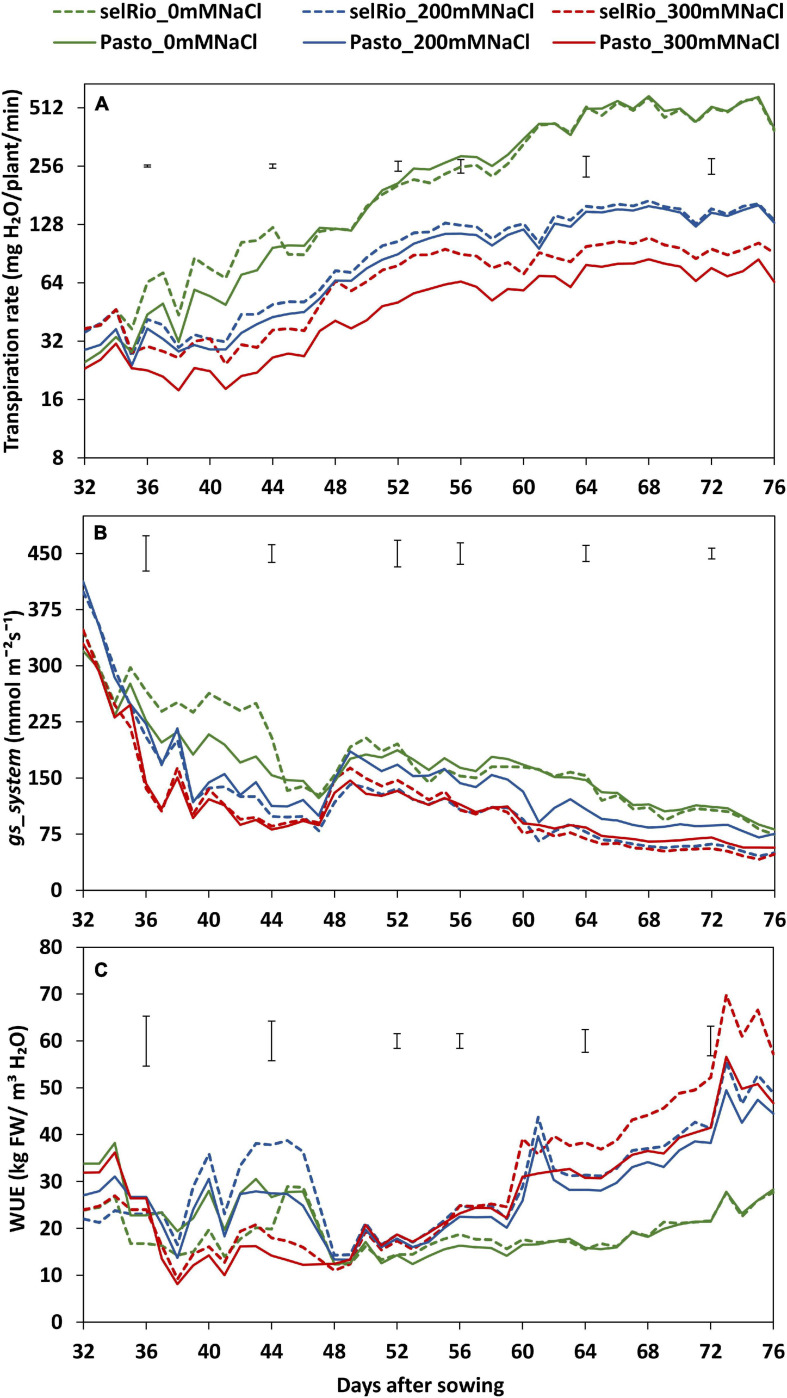
Water use parameters derived from Plantarray 3.0 data. **(A)** Average transpiration rate per day considering the hours of light received by the plants in the greenhouse. **(B)** Average stomatal conductance (*gs_system*) per day considering the hours of light received by the plants in the greenhouse. **(C)** Average whole-plant agronomic water use efficiency (WUE) per day considering the hours of light received by the plants in the greenhouse. Means of 4 plants. Error bars indicate the SEM (standard error of mean) from the ANOVA at a specific timepoint.

**FIGURE 4:**
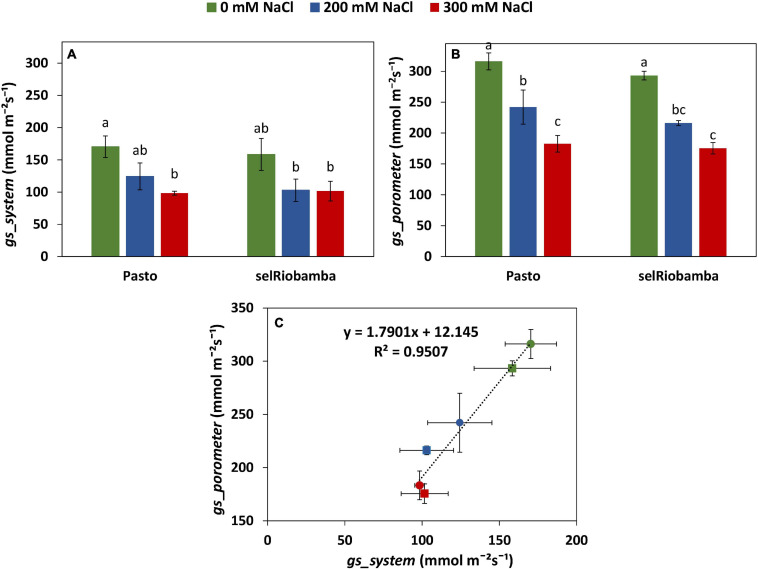
Comparison of the derived versus porometer stomatal conductance (*gs*). **(A)** *gs_system* derived from Plantarray data as the average *gs* between 12:30 to 13:15 at 58 DAS. **(B)** *gs_porometer* measured with a porometer from 12:30 to 13:15 at 58 DAS. **(C)** Correlation between the derived from Plantarray versus porometer *gs.* Means of 4 plants. Error bars indicate SE of individual means. Statistically significant differences (*p* ≤ 0.05) between any variety and salt treatment combination are shown with different letters.

**FIGURE 5:**
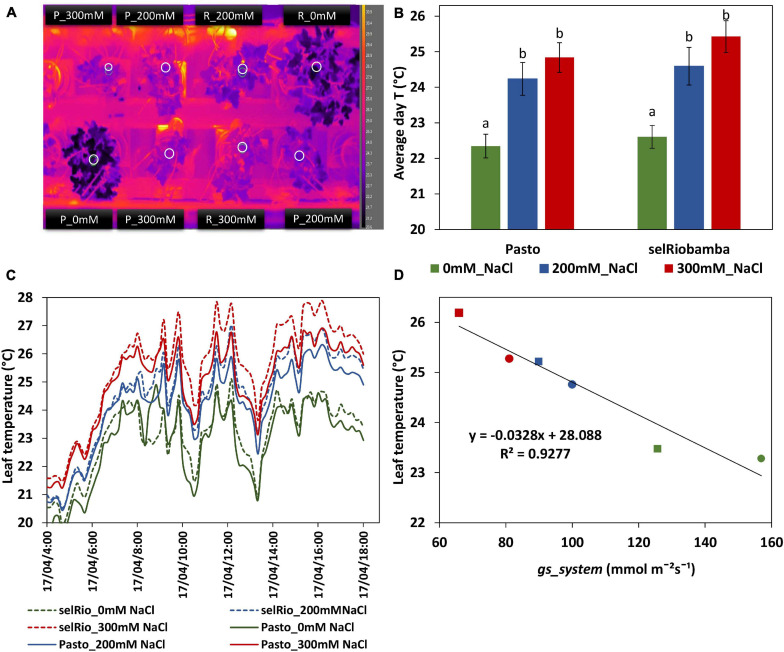
Leaf temperatures from thermal imaging for quinoa. **(A)** Thermal image obtained by a FLIR A655SC Thermal Camera. White circles indicate the regions used to determine the mean leaf temperature per plant. **(B)** Average leaf temperature during the day (07:00 AM–18:00 PM). Means of 7 consecutive days (59-65 DAS). Error bars indicate SE of individual means in the same plant. Statistically significant differences (*p* ≤ 0.05) between any variety and salt treatment combination are shown with different letters. **(C)** Fluctuation of leaf temperature during a day (63 days after sowing, 42 days after start of the stress). **(D)** Correlation between leaf temperatures and *gs_system* derived from Plantarray data.

### Ion Content Measurements

The ion content of young and old leaves, stems and roots was measured using Ion Chromatography (IC) system 850 Professional (Metrohm Switzerland). For this purpose, oven-dried tissues were ground to fine powder using a hammer mill with 1 mm sieve. Twenty-five mg per sample was ashed in a furnace at 550°C for 5 h. Ten ml of Milli-Q^®^ water was added to the ashes and these were shaken for 15 min at 5,000 rpm at 100°C. Prior to injection onto the IC system, samples were diluted 400 times with Milli-Q^®^. Ion contents were calculated as the amount of ions per unit of dry weight (mg ion g^–1^ dry mass) and the ion concentrations were estimated based on the water content of the tissue. The ratio K^+^/ Na^+^ was calculated based on mg K^+^/ mg Na^+^ content.

### Statistical Analysis

General analyses of variance (ANOVA) were performed to determine the significance of genotypic differences, salt treatment differences and their interactions (*p* < 0.05). The analyses were performed following a standard procedure for a linear mixed model, for which genotype and salt treatment were considered fixed effects and blocks random effects. The above-mentioned model was: *y*_*ijk*_ = μ + *b*_*k*_ + α_*i*_ + β_*j*_ + αβ_*ij*_ + *e*_*ijk*_, were *y*_*ijk*_ is the response variable, μ is the grand mean, α_*i*_ is the salt treatment effect, β_*j*_ is the genotype effect, αβ_*ij*_ is the genotype-by-salt interaction effect, *b_k_* is the block effect and *e_ijk_* is the residual error. Multiple comparison analyses were performed using Fisher’s protected least significant difference (LSD) test on genotype means. All statistical analyses were performed using the software Genstat 19th Edition (VSN International Hemel Hempstead, United Kingdom). The Treatment structure used in Genstat was Variety ^∗^ Salt concentration. The Block structure was plainly Blocks (four blocks laid out as A) pots 1-6, 7-12, 13-18, 19-24). Each block had one replicate pot of each treatment × variety combination. Each pot contained four plants (two for harvest point on DAS 47 and two for harvest point DAS 77). The pots were considered to be the experimental units. The two harvest time points of plants from the pots on the system were seperately analyzed with ANOVA as the residual variance depended on the time.

## Results

### General Salt Stress Response of the Plants

The effect of salt stress on the biomass and ion distribution in plant tissues of Pasto and selRiobamba was similar to previous evaluations (Jaramillo Roman et al., 2020). Two destructive harvests were carried out during this experiment. The first one at 47 DAS (11 days after the start of the stress) and the second one at 77 DAS (41 days after the start of the stress). After 11 days of salt stress, biomass was already significantly reduced and the average salt tolerance based on dry weight was 80% for the 200 mM NaCl treatment and 50% for the 300 mM NaCl treatment. At the second destructive harvest (6 weeks of salt stress), the impact of salinity on biomass was greater, with an average salt tolerance of 56% at 200 mM NaCl and 34% at 300 mM NaCl (Figure 1B). Both varieties were smaller but remained green and did not lose leaves despite the high salt treatments, but selRiobamba was significantly more salt tolerant than Pasto. The Na^+^, K^+^ and Cl^–^ concentrations were measured in roots, stems, old and young leaves at 77 DAS. The concentration of Na^+^ in selRiobamba showed an increasing gradient from roots to stem to leaves, and the concentration was slightly lower in young leaves compared to old leaves. Pasto on the other hand, showed lower [Na^+^] in leaves compared to roots and stems. The [Na^+^] of Pasto in young leaves at the 300 mM NaCl treatment was 156 mM, compared to 531 mM in selRiobamba (Supplementary Figure 1A). A similar trend was observed for the [Cl^–^] in different tissues. The highest concentration of Cl^–^ for selRiobamba was measured in leaves, while Pasto showed lower levels of Cl^–^ in young leaves compared to stems and roots (Supplementary Figure 1B). Salinity significantly decreased the [K^+^] of selRiobamba in all tissues. For Pasto, [K^+^] was not significantly affected by the 200 mM NaCl treatment and was significantly increased by an average of 20% in stems and young leaves at 300 mM NaCl (Supplementary Figure 1C). The K^+^/Na^+^ was higher in Pasto for all tissues and treatments.

### Monitoring Plant Growth and Transpiration Throughout the Season

The Plantarray phenotyping platform used in this study allowed us to monitor transpiration and biomass gain of plants continuously throughout the growing period (77 days). The cumulative water transpired by the plants is depicted in Figure 2A. Under control conditions, transpiration of Pasto and SelRiobamba was similar, in spite of their morphological differences (Pasto is a shorter variety and has higher leaf area per plant than selRiobamba). The salt treatment significantly affected the transpiration of plants. At 200 mM NaCl, transpiration was reduced by on average 60%. The more severe treatment of 300mM NaCl had a stronger effect on transpiration and also accentuated the differences between varieties. By the end of the experiment, the average cumulative transpiration per plant was 11 L in control conditions, while at 300 mM NaCl, transpiration was 66% lower for selRiobamba and 78% lower for Pasto (Figure 2B). The progressive accumulation of biomass was also monitored throughout the experiment (Figure 2C). Salinity had a significant effect on the fresh weight of plants already after four days (*p* < 0.001). Throughout the season, growth rates and biomass accumulation of both varieties were not significantly different under control conditions and were reduced by salinity. Biomass was more reduced in Pasto than selRiobamba. By the end of the experiment (after 6 weeks of salt treatment), the fresh biomass of selRiobamba was 35% decreased under 200 mM NaCl and 50% decreased under 300 mM NaCl, while Pasto biomass was 50 and 70% decreased under 200 and 300 mM NaCl, respectively (Figure 2D).

### Variation in Water Use Responses to Salinity Throughout the Season

Daily transpiration rate was calculated considering only the hours of light (Figure 3A). Salt-induced differences in the amount of water transpired were detected from the first day of salt treatment. Throughout the season, the transpiration rates were similar for the varieties under control conditions and under the lower salt treatment of 200 mM NaCl. However, under 300 mM NaCl, transpiration was clearly higher for selRiobamba. The differences in transpiration between salt treatments and varieties were significant. By the end of the experiment, the transpiration rate was reduced by 75% for selRiobamba under 300 mM NaCl and 82% for Pasto.

Stomatal conductance (*gs*) was calculated using transpiration rates and interpolated leaf area data as described in Materials and Methods. Salt had a significant effect on stomatal conductance already three days after the start of the salt treatment (Figure 3B). Under 200 mM NaCl, the *gs* for selRiobamba was 35% lower, while the *gs* for Pasto was 15% lower than control. Under 300 mM NaCl, the *gs* for both varieties was reduced by 35%.

Water use efficiency (WUE) at whole-plant level was calculated using Plantarray data as the ratio of cumulative biomass to cumulative water transpired. WUE was strongly influenced by the salt treatment throughout the growing period (Figure 3C). Shortly after the start of the salt treatment, WUE was lower at 300 mM compared to control and the 200 mM NaCl. However, a few days after the application of salt, WUE of the stressed plants exceeded the one of plants growing under control conditions. By the end of the experiment, WUE of both varieties at 200 mM NaCl was 56% higher than control. At 300 mM NaCl, Pasto WUE was increased by 60% and selRiobamba WUE by 75% compared to the controls.

### Plantarray Derived Versus Porometer Stomatal Conductance

The stomatal conductance derived from the Plantarray System data (*gs*_*system*_) was validated by comparing with the stomatal conductance measured with a steady state porometer (*gs*_*porometer*_) at 58 DAS (21 days after start of salt stress). Similar to *gs*_*system*_, salt-treated plants had significantly lower leaf *gs*_*porometer*_ than control plants and no significant differences were found between varieties (Figures 4A,B). A strong positive correlation of 0.95 was found between the *gs*_*porometer*_ and the *gs*_*system*_ (Figure 4C), indicating that the stomatal conductance calculations using the Plantarray data are valid and that the derived stomatal conductance is a reliable representation of stomatal behavior.

### Thermal Imaging as a Surrogate Estimation of Stomatal Conductance

Infrared thermography phenotyping was used as an additional tool to monitor plant stomatal responses to salt stress from 58-66 DAS (39-45 days after the start of the stress) (Figure 5). Leaf temperatures were higher in the leaves of stressed plants compared to the controls (Figures 5A–C). On average, the difference between control and stressed plants was 2°C, and the difference between the 200 and 300 mM NaCl treatments was about 1°C (Figure 2C). The daily leaf canopy temperature and the daily *gs* calculated from Plantarray parameters were highly correlated (R^2^=0.9277) (Figure 5D).

### Variation in Water Use Responses to Saline Conditions Throughout the Day

**FIGURE 6:**
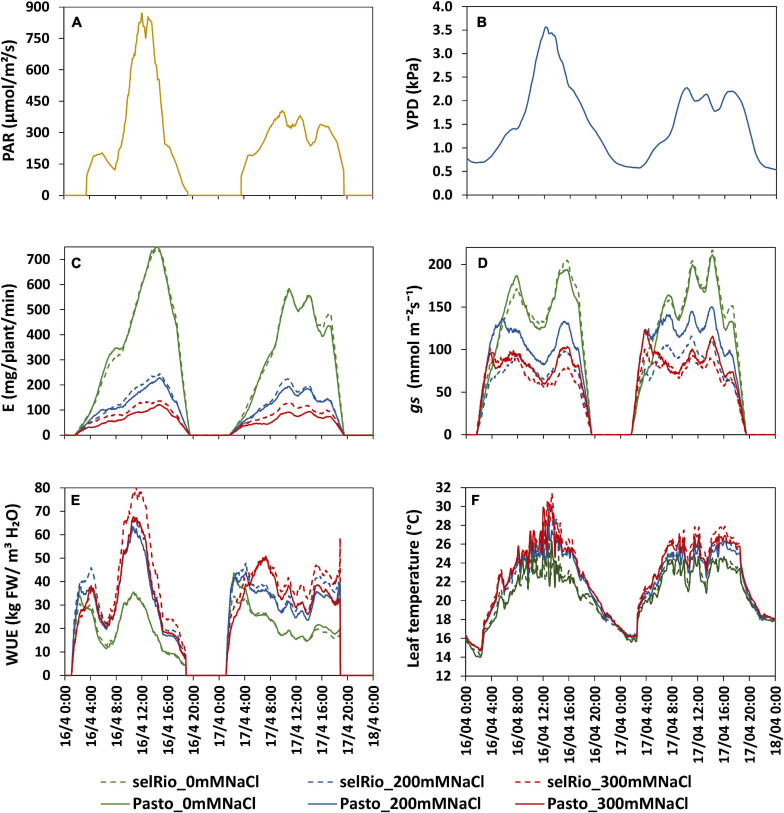
Daily patterns of experimental conditions measured by sensors and physiological components derived from Plantarray measurements. The influence of a bright (16/4/2019, 62 DAS, 41 days after the start of the stress) and cloudy day (17/4/2019, 63 DAS, 42 days after the start of the stress) are compared. **(A)** Light intensity. **(B)** VPD. **(C)** Transpiration rate (E). **(D)** Stomatal conductance (*gs*). **(E)** Water use efficiency (WUE). **(F)** Leaf temperature measured by a thermal camera.

The physiological traits measured in this study (transpiration rate, *gs*, WUE, leaf temperature) are influenced by environmental factors such as light intensity and VPD that vary between days and with the diurnal cycle. Figure 6 depicts the diurnal patterns of these parameters under control and saline conditions for two consecutive days (62-63 DAS, 41-42 days after the start of the stress). Light intensity and VPD showed considerable variation through the day and between days (Figures 6A,B). The first day had higher irradiance levels than the second day. Maximum PAR on the first day was 830 μmol m^−2^s^−1^ and occurred between 12:15 and 13:15 hrs. On the second day, the distribution of light was more homogenous during the day, and the maximum PAR recorded was 380 μmol m^−2^s^−1^. VPD patterns were similar to PAR patterns, with a maximum VPD of 3.5 kPa measured during the first day and of 2.2 kPa during the second day. The transpiration rate during the same two days showed clear differences between treatments, varieties, and days (Figure 6C). Transpiration under control conditions followed the pattern of PAR. On the first (bright) day, the transpiration rate at the highest PAR level of the day was 740 mg H_2_O/plant/min for both varieties, compared to a maximum rate of 580 mg H_2_O/plant/min measured on the second more cloudy day. Under salt stress, transpiration rate was stable during the day and not significantly different between days, indicating that under saline conditions, transpiration is less responsive to changes in PAR. Stomatal conductance showed an early morning peak that declined as VPD increased and reached a plateau during the late morning and midday hours (Figure 6D). The morning *gs* showed a peak earlier under salt conditions than under control conditions, which may be a strategy to maximize CO_2_ absorption despite the lower transpiration rate. WUE for plants under control conditions was very stable throughout the day on both days, and similar for both varieties (20 kg FW/m^3^ H_2_O) (Figure 6E). WUE was significantly higher in plants under stress conditions. At 200 mM NaCl, WUE was similar for both varieties. During the first day, a max WUE of 65 kg FW/m^3^ H_2_O was estimated around 11:00 AM (about one hour before the light irradiance and VPD max peaks recorded on the same day). During the second day with lower levels of irradiance, WUE values were also lower; the max WUE at 200 mM NaCl was 42 kg FW/m^3^ H_2_O. The varietal differences were even more pronounced under 300 mM NaCl treatment, and the relative differences between the varieties were also higher on bright days. At peak irradiation on day one, WUE of selRiobamba was about 79 kg FW/m^3^ H_2_O, while the WUE of Pasto at the same time was 15% lower. In line with *gs*, leaf temperatures of stressed plants were significantly higher than control plants during the whole day (Figure 6F). Differences in temperature were clear from the start of the light period. The highest differences in temperature were observed during the afternoon (13:00–18:00) when the average leaf temperature of control plants was 21°C and of stressed plants as high as 25°C. No significant temperature difference was observed between the 200 and 300 mM NaCl treatment. Leaf temperatures were different between a bright and cloudy day, especially for stressed plants. The maximum temperature registered for leaves of plants growing at 300 mM was 31°C at 13:00 PM, while the temperature of control plants at the same time was 24°C.

### Effect of Salt on the Photosynthetic Capacity of Quinoa

**FIGURE 7:**
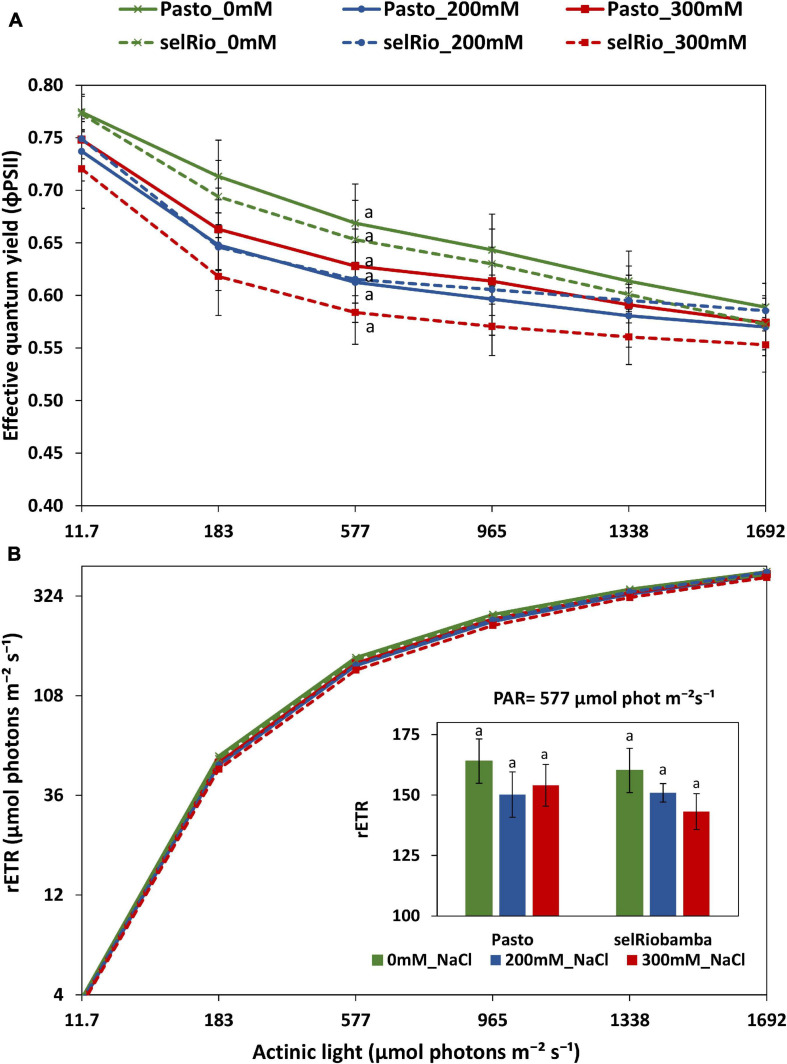
Derived parameters of a rapid light curve from light adapted detached young leaves of quinoa. **(A)** Effective quantum yield as a function of PAR. **(B)** Relative electron transfer rate as a function of PAR. Means of 4 plants. Error bars indicate SE of individual means. Statistically significant differences (*p* ≤ 0.05) between any variety and salt treatment combination are shown with different letters.

A rapid light response curve was recorded at 76 DAS to investigate the effect of salt on the photosynthetic capacity of quinoa plants, plotting effective quantum yield (φPSII) as a function of PAR irradiance (Figure 7A). φPSII provides an indication of the amount of energy used for photochemistry. At the lowest level of irradiance, φPSII has its maximum value, which for control was 0.77, indicative for a healthy leaf. No significant differences were found in the φPSII between treatments. At 183–965 μmol photons m^−2^s^–1^, the effect of salt was the most pronounced. For selRiobamba, φPSII was 7% lower at 200 mM NaCl and 11% lower at 300 mM NaCl. Pasto showed a 10% decrease at 200 mM NaCl but only a 5% decrease at 300 mM NaCl. φPSII multiplied by PAR gives a relative indication of the photosynthetic electron transport rate (ETR) (Figure 7B). Since φPSII is not linked to the amount of chlorophyll, the calculated parameter is the relative ETR (rETR) and is distinct from the ETR obtained from an oxygen-base P-E curve (Ralph and Gademann, 2005). The rETR rapidly increased with light intensity. However, the steady state was not reached with the maximum actinic light applied in this study (1692 μmol photons m^−2^s^−1^). The rETR of plants growing under salt treatment were slightly lower but not significantly different than control plants (Figure 7B).

### Functional Growth Analysis of Quinoa

**FIGURE 8:**
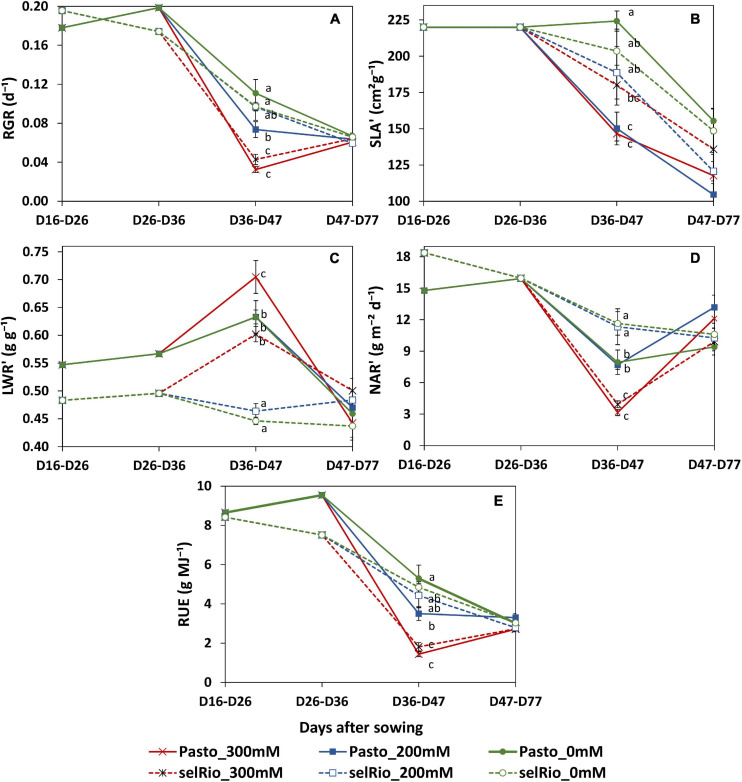
Relative growth components of quinoa throughout the growing period. **(A)** Relative growth rate (RGR). **(B)** Incremental specific leaf area (SLA’). **(C)** Incremental leaf weight ratio (LWR’). **(D)** Incremental net assimilation rate (NAR’). **(E)** Radiation use efficiency estimated by LINTUL mechanistic model. Means of 4 plants. Error bars indicate SE of individual means. Statistically significant differences (*p* ≤ 0.05) between any variety and salt treatment combination are shown with different letters.

During the course of this experiment, RGR and its components were monitored in three main periods: before the application of the stress, from the beginning of the stress until the first destructive harvest (36-47 DAS) and between the first and second destructive harvests (47-77 DAS). During the first phase of stress, salt significantly decreased RGR, especially in Pasto (Figure 8A). At 200 mM NaCl, selRiobamba RGR was similar to control, while Pasto’s RGR was already significantly lower (Figure 8A). The decrease in the RGR of Pasto at this time appeared to be mostly caused by a significant decrease in the specific leaf area (SLA) (Figure 8B), while the reduction in net assimilation rate (NAR) was mostly responsible for the reduced RGR of selRiobamba (Figure 8D). For both varieties, LWR was not significantly different between the control and 200 mM NaCl, but increased in the most severe treatment of 300 mM NaCl (Figure 8C). During the last period in which RGR analysis was performed (47-77 DAS), the RGR components were less affected by salt. Only the SLA was decreased by the salt treatment and, interestingly, NAR was even higher under salt stress than under control conditions for Pasto.

Radiation use efficiency (RUE) provides a measurement of the efficiency of a plant to use radiation energy for biomass production. In our experiment, the LINTUL crop model was used to estimate RUE as an integration of several physiological parameters. During the first period of growth after the application of the stress (36-47 DAS), RUE was significantly decreased by salt stress, and differences were found between varieties. For Pasto, RUE decreased by 33% at 200 mM NaCl and by 73% at 300 mM NaCl. For selRiobamba, RUE was only decreased at the highest salt concentration of 300 mM NaCl by 63% (Figure 8E).

## Discussion

We used the Plantarray phenotyping platform to gain insight in the salt response of two quinoa varieties, and in the consequences of different strategies with respect to transpiration, assimilation and growth. The impact of salt stress on the growth and physiological responses of the quinoa varieties Pasto and selRiobamba was similar as reported before (Jaramillo Roman et al., 2020). The plants remained green and were able to grow under salinity but dry biomass was strongly reduced by on average 44% at 200 mM NaCl and 66% at 300 mM after six weeks of the start of the salt treatment. Pasto and selRiobamba showed differences in their physiological responses to salinity, which resulted in a higher salt tolerance in selRiobamba than Pasto. Salt stress caused a reduction in RGR as a consequence of reduced a dry matter production from early stages of the stress. Similar reductions in quinoa growth caused by salt stress were reported before (Riccardi et al., 2014). The contribution of NAR and SLA to the decrease in RGR was different between varieties. The main cause of the reduced growth rate of Pasto was a decrease in SLA, which strongly indicates that the leaf area expansion rate of Pasto was relatively low, and leaf thickness increased. The SLA of selRiobamba was less affected; the main cause of the reduction of the growth rate of this variety under salinity appeared to be a lowered NAR, which is indicative of the photosynthetic capacity of the plant (Lambers and Poorter, 1992). The varieties also differed in ion uptake and distribution within plant tissues. In Pasto, the Na^+^ and Cl^–^ concentration in young leaves remained lower than the root medium, while in selRiobamba concentrations of 500 mM were measured for both ions, which points to a stronger shoot ion exclusion activity for Pasto. Differences in Na^+^ accumulation between quinoa varieties have been reported before (Kiani-Pouya et al., 2019). These differences can be attributed to varying degrees of how effective compartmentalization of Na^+^ in vacuoles can take place. While Pasto has to adapt physiological responses to reduce Na^+^ in the shoot, selRiobamba might tolerate higher Na^+^ concentrations due to a more effective vacuolar compartmentalization. As reported before, quinoa is recognized for its ability to retain or even increase K^+^ under salinity, especially in young photosynthetically active leaves (Hinojosa et al., 2018; Jaramillo Roman et al., 2020). Pasto and selRiobamba showed differences in K^+^ retention. In Pasto, [K^+^] in young leaves of salt stressed plants was 400 mM, 20% higher than in plants under control conditions, while in selRiobamba, [K^+^] was 50% reduced under the 300 mM NaCl treatment. The energetic cost of K^+^ retention under saline conditions is high: 1–2 mol ATP is needed for the retention of 1 mol of K^+^ (Rubio et al., 2020). For this reason, retaining K^+^ under salinity has been described as a ‘metabolic switch’, in which a larger amount of ATP is redirected to adaptive traits to salt stress (Rubio et al., 2020). Our results indicate that Pasto allocated more resources toward this adaptation, but this may have come at a metabolic cost, reflected in the higher reduction of biomass under salt stress (Figure 2).

### Whole-Plant Adaptations to Salt Stress

**FIGURE 9:**
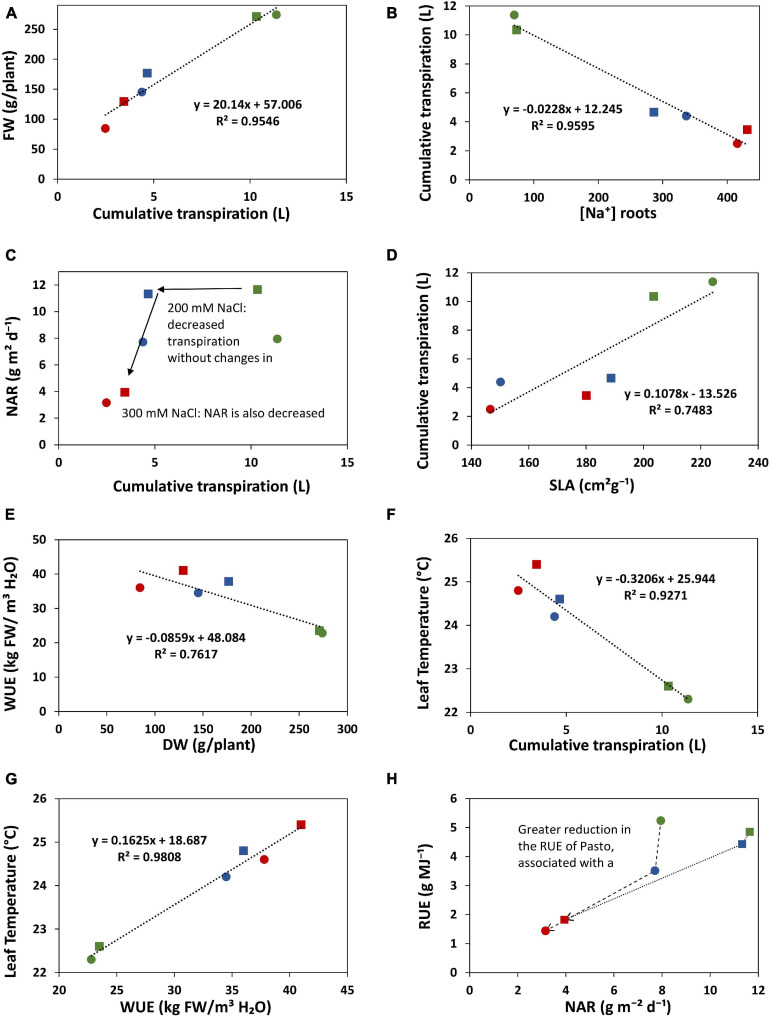
Relations between some of the physiological traits analyzed in this study. The values of selRiobamba are indicated by squares and of Pasto by circles. Level of stress is indicated by color; green: no-salt, blue: 200 mM NaCl, red: 300 mM NaCl. **(A)** Cumulative transpiration vs. fresh weight of plants. **(B)** [Na^+^] in roots vs. cumulative transpiration of plants. **(C)** Cumulative transpiration of plants vs. net assimilation rate (NAR). **(D)** Specific leaf area (SLA) vs. cumulative transpiration of plants. **(E)** Dry weight of plants vs. water use efficiency. **(F)** Cumulative transpiration vs. leaf temperature. **(G)** Water use efficiency vs. leaf temperature. **(H)** Net assimilation rate (NAR) vs. radiation use efficiency (RUE).

Transpiration was strongly reduced by salinity. Under the 200 mM NaCl treatment, cumulative transpiration was 60% reduced for both varieties. The 300 mM NaCl treatment strengthened this reduction and the differences between varieties; cumulative transpiration was 66 and 80% reduced in Pasto and selRiobamba, respectively. Cumulative transpiration had a strong positive correlation with the fresh weights of the plants (Figure 9A). However, under 200 mM NaCl, Pasto had a stronger reduction in biomass than selRiobamba while transpiration was similarly reduced. It is possible that while the available resources (water, CO_2_) in both varieties were similar, assimilates were less allocated to biomass production in Pasto, and more directed toward salt tolerance responses (morphological adaptations like decrease in SLA, Na^+^ and Cl^–^ exclusion, K^+^ retention, among others). Transpiration was significantly correlated to [Na^+^] and [Cl^–^] in the roots (Figure 9B) but not in young leaves. In addition, Na^+^ and Cl^–^ concentrations in leaves of Pasto were lower than selRiobamba, while the transpiration rate in both varieties was similar. This indicates that the ion concentrations in young leaves may be more determined by ion exclusion mechanisms (mainly at xylem loading) than by the transpiration rate of the plants. We examined the effect of the reduction of transpiration on the growth rate and the RGR components. Despite the 60% reduction in transpiration, the 200 mM NaCl treatment did not affect NAR, which means the photosynthetic rate was not affected by this for quinoa mild salinity level (Figure 9C). Transpiration had a positive correlation with SLA (Figure 9D). The morphological adaptation of reduced leaf expansion and thicker leaves reduced the total surface available for water loss, which agrees with the lower transpiration of Pasto, especially under 300 mM NaCl.

Interestingly, selRiobamba had a smaller reduction in total plant transpiration than Pasto, but its stomatal conductance (transpiration per unit leaf area, standardized for VPD) was more reduced than that of Pasto. Our results indicate that this may be explained by the stronger reduction in leaf expansion of Pasto. We argue that this might be an important difference between the salt stress response of these quinoa varieties. Pasto lowers total transpiration by a decreased leaf area (without strong control of stomata) while selRiobamba appears to control stomatal aperture to minimize water loss and optimize transpiration.

**FIGURE 10:**
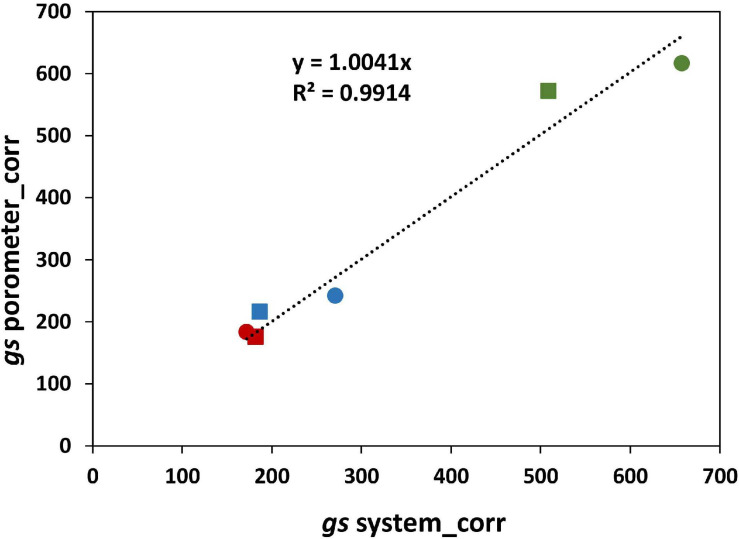
Correlation between *gs* porometer measurements corrected for the abaxial and adaxial sides of the leaves (*gs*_*porometer_corr*_) and the *gs* from the system after correcting for the effect of the boundary layer resistance (*gs*_*sysyem_corr*_).

The effect of salinity on the stomatal conductance calculated from Plantarray data (*gs*_*system*_) was comparable to the effect on the stomatal conductance measured with a porometer (*gs*_*porometer*_) (Figure 4), validating the *gs*_*system*_ calculations. However, the *gs*_*system*_ values were approximately 46% lower than the *gs*_*porometer*_ values under all treatments. This seems counterintuitive as the *gs*_*system*_ was derived from the total leaf transpiration (transpiration from both the abaxial and adaxial side of leaves), while *gs*_*porometer*_ represents the conductance only from the abaxial side of a leaf. We tested the relative contribution of abaxial and adaxial stomatal conductance to the total stomatal conductance with plants grown at 0 and 300 mM NaCl in a separate experiment. At 0 mM, the adaxial *gs* was not significantly different from the abaxial *gs*; the ratio of adaxial to abaxial *gs* was 1.02 (Supplementary Table 2). At 300 mM, however, this ratio was much lower than 1 (0.76). We used this information to correct the whole plant *gs* calculated from porometer *gs* values for the relative contribution of the abaxial and adaxial sides of the leaves. Another parameter that needs to be considered when comparing *gs*_*porometer*_ and *gs*_*system*_ is the boundary layer resistance. The *gs*_*porometer*_ data is not affected by the boundary layer resistance (McDermitt, 1990). Yet the influence of boundary layer resistance in plants growing in the greenhouse might considerably decrease whole-plant conductance (Katsoulas et al., 2007), and the effect of the boundary layer resistance (*gb*) is not considered in the calculation of the *gs*_*system*_. The *gs_*system*_*, which in fact is the total conductance, includes *gs* as well as *gb*, and is equal to 1/(1/*gs*+ 1/gb). Here, *gs* is the stomatal resistance from both sides of the leaves combined and *gb* is the boundary layer resistance. Therefore, the whole plant *gs* was used to estimate a single boundary layer resistance for this experiment (230 mmol/m^2^/s). The boundary layer resistance in greenhouses varies from 200 to 2,000 mmol/m^2^/s depending on the wind speed and the size of leaves (Kimura et al., 2020). The *gb* calculated in our experiment corresponds to a very low wind speed of 0.05 m/s, which is in agreement with the conditions in our greenhouse. The *gs*_*system*_ values corrected for the boundary layer resistance (*gs*_*sysyem_corr*_) are highly comparable to *gs*_*porometer*_ values corrected for both sides of the leaf (*gs*_*porometer_corr*_) (Figure 10).

WUE can be defined and measured in different ways; it can be an instantaneous measurement of the ratio of the photosynthetic rate and the transpiration rate, or a productivity measurement of the ratio of biomass accumulation and water use over a period of time (Leakey et al., 2019). The Plantarray system provides a platform to continuously monitor changes in WUE that result from dynamic interactions between water use and biomass gain by the plants. Thus, WUE can be studied as a dynamic process more than a productivity indicator. In our study, salinity significantly increased the WUE of quinoa. It should be noted that the WUE calculated from Plantarray data is expressed as kg Fresh Weight/m^3^ H_2_O, and not dry weight; however, the differences in WUE caused by the salt treatment in quinoa are not a result of a lowered water content in the leaves of salt stressed plants, since the differences in the dry matter content between treatments were negligible (data not shown). A main goal of breeding for salt or drought stress tolerance is to improve the WUE of plants, but only as long as this also supports greater productivity under the stress conditions (Leakey et al., 2019). High values of WUE are typically observed when stomatal conductance is lower that the potential maximum for a genotype, which also results in reduced growth (Yoo et al., 2009). Therefore, higher values of WUE are often associated with smaller plants, lowered growth and low crop production (Blum, 2009). By continuously monitoring WUE and parameters that might affect the WUE (evaporation, transpiration rate, biomass gain, stomatal conductance, leaf area, and environmental parameters such as VPD) we might be able to identify whether the increase of WUE in a particular genotype is mostly associated with lower water loss, or whether certain adaptations contribute to increase productivity and maximizing the efficiency of water use. Both Pasto and selRiobamba showed higher WUE under salinity; however, with similar amounts of water transpired, more biomass was produced by selRiobamba (Figure 9E). The increased WUE might therefore be a favorable trait for the productivity of selRiobamba, and more of a water-saving strategy for Pasto; the causes of the increase of WUE in both varieties might be associated with different physiological mechanisms that should be further explored.

One of the greatest advantages of the Plantarray system is the temporal resolution of the measurements that enables to monitor water use responses not only to environmental variation throughout the growing cycle, but also to diurnal variations of environmental parameters like light irradiance and atmospheric VPD. The day-to-day patterns of transpiration, *gs* and WUE were compared between two consecutive days that showed different levels of VPD and light irradiance (Figure 6). The relative differences in the transpiration rate between days were higher under control than under salt stress. Reducing transpiration is a common adaptation to saline conditions. Photosynthesis is primarily limited by CO_2_ uptake, while it is affected by light availability to a much lower extent (Flexas et al., 2004). Assuming the photosynthetic machinery is saturated, the additional water transpired under control conditions in a day with higher irradiation will be either wasted or used for canopy cooling purposes, but will not be associated with higher biomass synthesis. Under saline conditions, the plant cannot afford to waste water. Therefore, a tighter control of transpiration rate by quinoa under salt stress is necessary. Stomatal conductance was influenced by the fluctuations in VPD during the day, which has been identified as a “patchy” stomatal behavior (Buckley, 2005). The daily *gs* is depressed at maximal VPD (midday), and a high *gs* is observed in the early morning, when light irradiance increases and VPD is still low (Gosa et al., 2019). When VPD is high, evaporation from the leaves is high as well, so a strict control of stomatal opening at high VPD might be an additional strategy to enhance CO_2_ uptake without excessive loss of water. The continuous monitoring of gs indicated that quinoa has a strict control in stomatal opening, which might be even increased under salinity. However, the total amount of water that could be saved throughout the growth cycle by the temporal control of stomatal opening needs to be estimated.

Infrared thermography was used to monitor salinity-induced changes in leaf temperature. Leaf temperature has been considered a proxy for *g*_*s*_ (Hackl et al., 2012; Ivushkin et al., 2018), and canopy thermography was also used as an indicator of salinity stress in quinoa (Ivushkin et al., 2019). The surface of a leaf is cooled by evaporation, so a strong correlation exists between the cooling of the leaves with transpiration rate and stomatal opening. In our study, canopy temperature had a strong negative correlation with *g*_*s*_, transpiration, biomass, and growth, and a positive correlation with water use efficiency, Na^+^ and Cl^–^ content in young leaves (Figures 9F,G). Salinity significantly increased the leaf temperature by 2°C at 200 mM NaCl and 2.7°C at 300 mM NaCl. Genotype-specific responses could also be identified using infrared thermography (Figure 5). The leaf temperatures of selRiobamba were slightly but not significantly higher than Pasto. Previously, we pointed out that Pasto had a lower transpiration rate than selRiobamba, and suggested that this was achieved by decreasing SLA rather than decreasing *gs*. An additional advantage of this adaptation could be that water loss is reduced without compromising the cooling system of leaves. In this way, significant differences were found in the transpiration rate and *gs* of Pasto and selRiobamba, without significant differences in leaf temperatures. Based on our measurements, leaf temperature has the potential to be used as a proxy to *gs*, also to WUE; the diurnal pattern of WUE was very well followed by the leaf temperature pattern, and both traits were highly correlated (r = 0.99).

Reduced stomatal conductance under salinity stress is an important determinant for reduced photosynthetic activity. However, other non-stomatal photosynthesis-limiting factors might also play a role when plants face salt stress. Chlorophyll fluorescence was used to measure the response of photosynthetic parameters to salinity. Rapid light curves provide information on the saturation characteristics of electron transport, as well as the overall photosynthetic performance of a plant over a wide range of ambient light intensities (Ralph and Gademann, 2005). It was previously reported that net apparent photosynthesis activity (A_N_) and the internal CO_2_ concentration at PAR levels higher than 500 μmol photons m^−2^s^−1^ were significantly reduced in quinoa by a salt treatment of 250 mM NaCl, while photochemical parameters, light compensation point and maximum apparent photosynthetic quantum yield were not affected (Becker et al., 2017). In our experiment, φPSII and rETR as a function of irradiance were more impacted by salt treatment than the Fv/Fm ratio of dark-adapted leaves, but no significant differences were found in these parameters between treatments. However, at lower light levels in the range of 183 to 965 μmol photons m^−2^s^−1^ the maximum rate of photosynthesis was lowered (similar to the LICOR measurements by Becker et al. (2017)). In a crop situation, most leaves are exposed to medium light levels, which means that a stronger effect of salt (at 200 and 300 mM NaCl) on the maximal photosynthesis rate may be experienced. Even though the φPSII was not significantly different between treatments and varieties, it was slightly lower for selRiobamba than Pasto, and Pasto had a higher φPSII under 300 mM NaCl than 200 mM NaCl. It is possible that the higher φPSII of Pasto is also related to its lower SLA. Thicker leaves likely have a higher density of Rubisco and chlorophyll per unit of leaf area, thus their photosynthesis rate might increase (Terashima et al., 2011).

### Varietal Differences in Responses to Salt Stress From a Resource Use Perspective

The differences in morphological and physiological traits associated with the use of water, energy and assimilates of Pasto and selRiobamba in response to salinity indicate that these varieties may use different strategies to cope with salt stress. Pasto had the lowest [Na^+^] and [Cl^–^] in the leaves and the highest [K^+^]. In addition, it had the highest reduction in transpiration and the lowest growth rate, which was mostly associated with a decreased SLA (Figure 9H). These traits are in line with a “conservative growth” strategy aimed at survival that is reflected in reduced growth and a higher investment in metabolically expensive stress tolerance traits (e.g., K^+^ retention) that constitute a trade-off to growth. A genotype like Pasto with its reduced growth rate and higher K^+^ retention (as observed in our study) will conserve water in the soil on the one hand (less transpiration and therefore less increase in salinity) and will be able to tolerate a higher increase in salinity at the end of the growing season. In selRiobamba transpiration was less reduced by saline conditions, Na^+^ and Cl^–^ accumulated to higher levels in the shoots and [K^+^] in leaves was maintained, but did not increase as much as in Pasto. SelRiobamba appeared to follow an “acquisitive growth” strategy aimed at continued growth in spite of the stress.

The relation between allocation of resources, functional traits and stress tolerance has been extensively studied (Reich et al., 2003; Reich, 2014; Balachowski and Volaire, 2018). Fast growers or “acquisitive-growth” plants are normally more productive under moderate stress, but under prolonged or severe stress they might exhaust the limitedly available resources, risking plant failure and death. On the other hand, slow growers or “conservative growth” plants are typically more penalized under moderate stress, but better survivors under more severe conditions.

Our results suggest that a conservative or acquisitive growth strategy of a quinoa variety is influenced by the severity and duration of the stress in addition to a genetic component. The conservative growth response of Pasto serves to protect tissues to prolonged and severe salinity and Pasto would therefore be better able to survive such conditions than selRiobamba. However, the acquisitive growth strategy of selRiombamba was more favorable in the conditions of this experiment (relatively mild for quinoa and until flowering stage), demonstrated by a higher growth rate and higher accumulation of green biomass. Our study shows a method of analyzing genetic differences in response to salinity and this may provide a selection tool for breeding. Further validation of the hypothesized genetic differences in the field will help to evaluate the best way to implement these growth strategies as selection criteria in breeding programs.

The ability of quinoa to adapt strategies is to some extent also reflected in the results of this study; while at 200 mM NaCl Pasto’s transpiration rate was similar to selRiobamba, the higher stress level changed Pasto’s behavior to a more conservative growth strategy. Possibly, quinoa can be called a facultative halophyte because of the ability to switch from an acquisitive growth to a conservative growth strategy when stress becomes severe and less resources are available, and Pasto and selRiobamba differ in the salinity threshold that flips this switch. Accurate and high-resolution phenotyping platforms like the Plantarray system are highly useful tools to distinguish physiological differences between both strategies, and to identify genetic variation that can be used to improve quinoa yields in a broad range of saline environments.

### Future Perspectives of Functional Phenotyping in Abiotic Stress Tolerance Research

Several reports have examined the influence of salt stress on physiological parameters related to water and carbon fluxes, photosynthesis and ion contents in quinoa. However, extrapolating data from limited timepoint measurements on single leaves (e.g., gas exchange rate, rate of net photosynthesis) to the growth cycle of a crop is not straightforward (Harris et al., 2010; Morton et al., 2019). This study shows the potential of the implementation of high throughput functional phenotyping in the understanding of complex physiological responses to salt stress. The Plantarray system is scalable, which means that a high number of plants and genotypes could potentially be screened simultaneously. The possibility to scan entire mapping populations opens up the possibility of identifying genetic determinants underpinning differences in traits such as water use efficiency or the diurnal control of stomatal conductance. The system can for instance be complemented with digital imaging systems that monitor leaf expansion and estimate leaf area, which will produce even more accurate measurements of stomatal conductance. In addition, the high-resolution continuous monitoring of growth and transpiration provides valuable data for the development and improvement of crop growth models. We used the mechanistic crop model LINTUL to integrate several physiological processes for an estimation of the radiation use efficiency (RUE) and to analyze the impact of salinity on the RUE of quinoa (Spitters and Schapendonk, 1990; Sinclair and Muchow, 1999). We estimated average RUE values of 5 gDW/MJ under control conditions. Previous studies in quinoa reported a significantly lower RUE (1.4–1.75 gDW/MJ) (Ruiz and Bertero, 2008; Razzaghi et al., 2012). This difference may be attributed to different growing conditions and the time of the measurements. High RUE values (3–5 gDW/MJ) are often reported in plants growing under controlled conditions due to the high proportion of diffuse radiation inherent to glasshouses and the lower daily incident radiation that might induce higher photosynthetic efficiency (Cabrera-Bosquet et al., 2016). In our conditions, RUE was significantly reduced by salt stress, which is in contrast with previous studies in which no differences in RUE were found even with a salt treatment of 40 dS/m (Razzaghi et al., 2012). Razzaghi et al. (2012) estimated RUE in a field trial where the average light level experienced by the leaves is much lower. In those conditions the initial light use efficiency determines RUE. In our conditions, where the LAI remained much lower than in a crop situation, most leaves experienced higher light levels than in a crop. This will explain the lower overall RUE estimated under stress conditions. Although genetic improvement of RUE has been suggested as a way to increase yield, few studies have explored its genetic variation, probably due to technical difficulties in the estimation of this parameter (Cabrera-Bosquet et al., 2016). Our results suggest that using the Plantarray system data as input for growth models may be a viable strategy for crop improvement based on RUE. High throughput and high-resolution technologies thus enable the dissection of plant growth and water consumption into very specific parameters that could constitute novel targets for the improvement of abiotic stress tolerance of crops.

## Data Availability Statement

The raw data supporting the conclusions of this article will be made available by the authors, without undue reservation.

## Author Contributions

VJR performed the experiments, analyzed the data, and wrote the original draft of the manuscript. RZ provided access to the phenotyping facilities from the Netherlands Plant Eco-phenotyping Centre (NPEC). JP carried out the thermal imaging experiments. RGFV helped to conceptualize the study, discussed the outcomes, and reviewed and edited the manuscript. CGL coordinated and supervised the research, conceptualized the experimental design, guided the discussion of the outcomes, and reviewed and edited the manuscript. ENL conceptualized the experimental design, performed the experiments, derived the physiological data, coordinated and supervised the research, guided the discussion of the outcomes, and reviewed and edited the manuscript. All the authors read and approved the manuscript.

## Conflict of Interest

The authors declare that the research was conducted in the absence of any commercial or financial relationships that could be construed as a potential conflict of interest.

## Publisher’s Note

All claims expressed in this article are solely those of the authors and do not necessarily represent those of their affiliated organizations, or those of the publisher, the editors and the reviewers. Any product that may be evaluated in this article, or claim that may be made by its manufacturer, is not guaranteed or endorsed by the publisher.

## Supplementary Material

Supplementary Figure 1 Ion contents in tissues of quinoa at different levels of salinity 77 DAS (41 days after the start of salt stress). **(A)** [Na^+^] in different tissues of quinoa at different levels of salinity. **(B)** [Cl^–^] in different tissues of quinoa at different levels of salinity. **(C)** [K^+^] in different tissues of quinoa at different levels of salinity. Means of 4 plants. Error bars indicate SE of individual means. Statistically significant differences (*p* ≤ 0.05) between any variety and salt treatment combination (within each tissue) are shown with different letters.

Supplementary Table 1 Timetable of the different measurements/treatments included in this study.

Supplementary Table 2 Comparison between *gs* measured with a porometer in the abaxial and the adaxial side of leaves of quinoa plants growing under control conditions (0 mM NaCl) or under salt treatment (300 mM NaCl) at 64 DAS (35 days after the start of salt treatment).
