# Supplementary material for: High-Resolution Analysis of Growth and Transpiration of Quinoa Under Saline Conditions
Source: Front Plant Sci. 2021 Aug 5;12:634311. doi: 10.3389/fpls.2021.634311 (PMC8376478; doi:10.3389/fpls.2021.634311)

Supplementary Figure 1. Ion contents in tissues of quinoa at different levels of salinity 77 DAS (41 days after the start of salt stress). A) $[Na^+]$ in different tissues of quinoa at different levels of salinity. B) $[Cl^-]$ in different tissues of quinoa at different levels of salinity. C) $[K^+]$ in different tissues of quinoa at different levels of salinity.Means of 4 plants. Error bars indicate SE of individual means. Statistically significant differences ($p \leq 0.05$) between any variety and salt treatment combination (within each tissue) are shown with different letters.

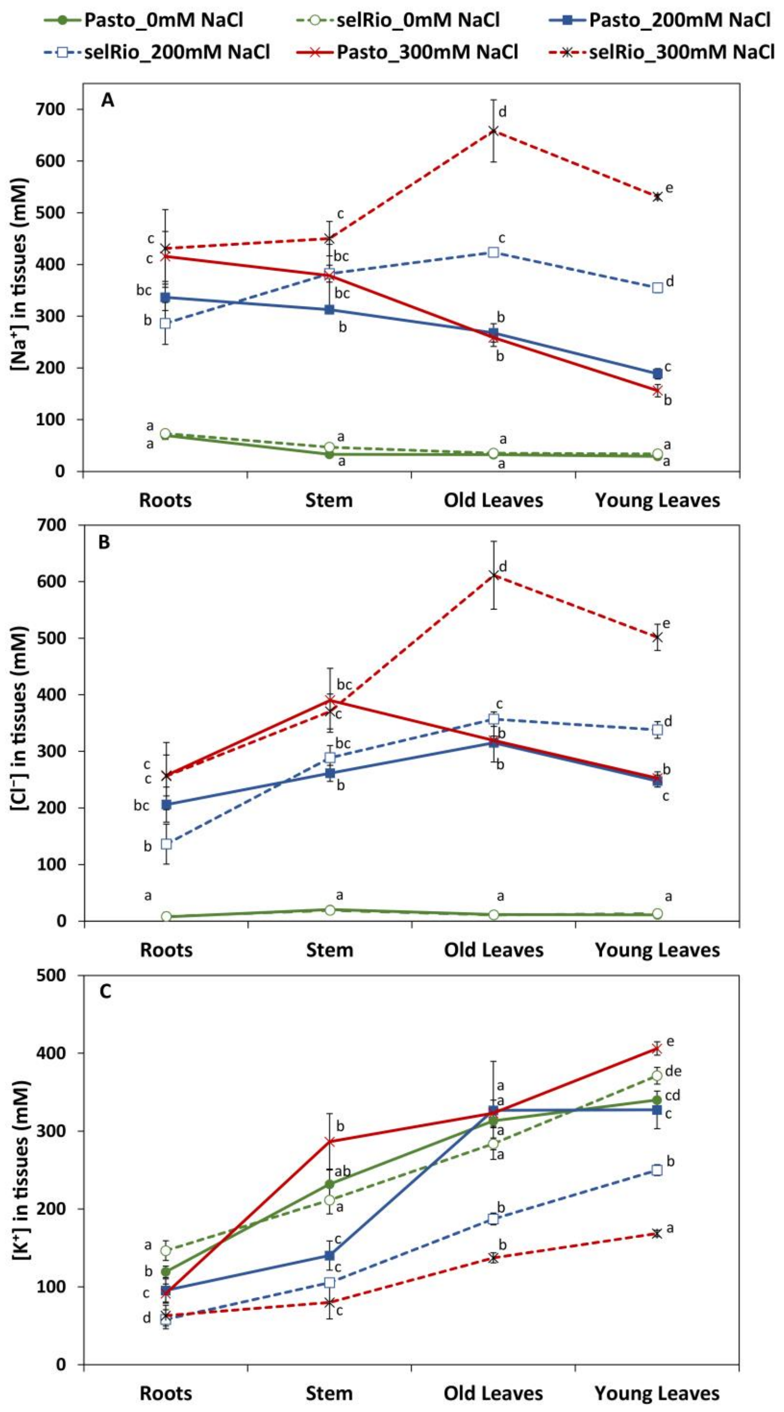

Supplement: Supplementary Figure 1 — Ion contents in tissues of quinoa at different levels of salinity 77 DAS (41 days after the start of salt stress). (A) [Na+] in different tissues of quinoa at different levels of salinity. (B) [Cl–] in different tissues of quinoa at different levels of salinity. (C) [K+] in different tissues of quinoa at different levels of salinity. Means of 4 plants. Error bars indicate SE of individual means. Statistically significant differences (p ≤ 0.05) between any variety and salt treatment combination (within each tissue) are shown with different letters. [file Image_1.pdf]
