# Supplementary material for: High-Resolution Analysis of Growth and Transpiration of Quinoa Under Saline Conditions
Source: Front Plant Sci. 2021 Aug 5;12:634311. doi: 10.3389/fpls.2021.634311 (PMC8376478; doi:10.3389/fpls.2021.634311)
Supplement: Supplementary Table 1 — Timetable of the different measurements/treatments included in this study. [file Table_1.docx]

**Supplementary Table 1.** Timetable of the different measurements/treatments included in this study.

| Activity/Measurement | Days after sowing (DAS) |
| --- | --- |
| Sowing | 0 |
| Transplant seedlings into pots | 16 |
| Moving pots into Plant Ditech system | 26 |
| Salt stress start | 36 |
| Harvest of 2 plants per pot | 47 |
| Validation of *gs* estimation with a portable porometer | 58 |
| Thermal imaging of plants | 58-66 |
| Analysis of water use components throughout the day | 62-63 |
| Chlorophyll fluorescence measurements | 76 |
| End of the experiment | 77 |
