# Supplementary material for: High-Resolution Analysis of Growth and Transpiration of Quinoa Under Saline Conditions
Source: Front Plant Sci. 2021 Aug 5;12:634311. doi: 10.3389/fpls.2021.634311 (PMC8376478; doi:10.3389/fpls.2021.634311)
Supplement: Supplementary Table 2 — Comparison between gs measured with a porometer in the abaxial and the adaxial side of leaves of quinoa plants growing under control conditions (0 mM NaCl) or under salt treatment (300 mM NaCl) at 64 DAS (35 days after the start of salt treatment). [file Table_2.docx]

**Supplementary Table 2.** Comparison between *gs* measured with a porometer in the abaxial and the adaxial side of leaves of quinoa plants growing under control conditions (0 mM NaCl) or under salt treatment (300 mM NaCl) at 64 DAS (35 days after the start of salt treatment). Means of 6 plants.
